# chewR: An Open‐Source R Package for Quantifying Masticatory Performance With Applications in Oral Processing Research

**DOI:** 10.1111/jtxs.70112

**Published:** 2026-09-01

**Authors:** John W. Long, Elsbeth Akanzinge, John E. Hayes

**Affiliations:** ^1^ Department of Food Science The Pennsylvania State University University Park Pennsylvania USA; ^2^ Department of Nutritional Sciences The Pennsylvania State University University Park Pennsylvania USA; ^3^ Sensory Evaluation Center The Pennsylvania State University University Park Pennsylvania USA

**Keywords:** chewing efficiency, chewing performance, eating rate, image analysis, masticatory performance, open‐source software, Oral processing, two‐color gum test

## Abstract

Masticatory performance is a measure of oral physiology that affects food breakdown and eating behavior. Existing methods to quantify masticatory performance rely on labor‐intensive procedures (e.g., sieving expectorated samples), or proprietary software (e.g., ViewGum), limiting adoption and reproducibility. In this cross‐sectional methods validation study, we: (1) examine the convergent validity of chewR, a user‐friendly R package that quantifies masticatory performance in a 2‐color gum mixing task via automated image analysis, and (2) examine how chewR based masticatory performance measures relate to other oral processing behaviors. Adults (*n* = 47, 601% female) completed a laboratory visit with a 2‐colored gum mixing task, stimulated salivary flow, and eating rate objectively measured using Tang's carrot test. Chewed gum samples were flattened with a standardized method, scanned, and resulting images were analyzed using ViewGum and chewR. ViewGum generates a score based on the gum color mixing, with lower scores indicating greater masticatory performance. chewR produces a raw score based on gum color mixing; from this, a transformed chewR Masticatory Performance Score was calculated, with higher values indicating greater masticatory performance. chewR measures showed strong convergent validity with ViewGum Score: the raw chewR score and transformed chewR Masticatory Performance Score were each strongly correlated with ViewGum Score (*r*'s = ±0.81, *p* < 0.00001), with signs in expected directions. Regarding predictive validity, the transformed chewR Masticatory Performance Score was weakly associated with carrot eating rate (*r* = 0.12), self‐reported eating rate (*r* = 0.20), and stimulated salivary flow (*r* = 0.21); the same associations were similarly weak for ViewGum Score. These findings support chewR as an open‐source alternative to ViewGum to quantify masticatory performance in a gum mixing task.

## Graphical abstract

Masticatory performance is a functional measure of oral physiology that may affect food breakdown and/or eating behavior. Existing methods are labor intensive or require proprietary software; here we describe a new open‐source package, chewR, to measure masticatory performance.

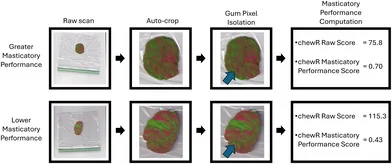

## 1 Introduction

Increased incidence of chronic disease has been fueled by characteristics of our eating environment that promote overconsumption (Kirk et al. 2010). Such environments are characterized by highly palatable foods that promote faster consumption by reducing oral processing demands through properties such as texture (Forde et al. 2020). This presents a significant public health concern given consistent associations between faster eating rates and greater food intake (Forde et al. 2026; van Bruinessen et al. 2026). The robust nature of this finding supports growing interest in oral processing behaviors as a potential mechanistic link between food properties and eating behavior. Within this framework, masticatory performance is an oral processing behavior that reflects how effectively food is broken down during mastication (Gonçalves et al. 2021). Individual differences in masticatory performance could potentially explain variation in oral processing that contribute to faster consumption and greater energy intake.

Numerous methods have been developed to measure masticatory performance, including particle size analysis of chewed specimens, mechanical and muscular measures of mastication, and image‐based analyses of chewed foods or test materials (Gonçalves et al. 2021). While many of these methods have clear utility, they lack scalability due to their reliance on specialized equipment and/or time‐intensive procedures. Among these methods, the two‐color gum mixing task is non‐invasive, inexpensive, and easy to standardize. In this task, participants chew a piece of gum composed of two distinct colors for a fixed number of chews (Gonçalves et al. 2021). The chewed gum is then flattened, scanned, and analyzed via image‐based methods (Halazonetis et al. 2013; Schimmel et al. 2015). Masticatory performance is then quantified from the resulting color distribution of gum where more uniform color mixing indicates more effective chewing. That is, efficient chewing results in more uniform color distribution, whereas less efficient chewing results in greater color segregation. This task was recently used to study broader oral processing mechanisms by linking masticatory performance to bolus properties and salivary uptake (Zannidi et al. 2025). Other work has aimed to streamline some of the cumbersome aspects of this task by demonstrating that images captured with a smartphone can be used for analysis of chewed gum samples; however, while smartphone images are convenient, the authors note that flatbed scanners remain the current gold standard (Buser et al. 2018; Fankhauser et al. 2020; Halazonetis et al. 2013). Despite the utility of imaging methods, they often rely on proprietary software and manual image‐processing steps, which may limit scalability, reproducibility, and accessibility. Such limitations highlight a need for open‐source tools that facilitate standardized and reproducible assessment of masticatory performance.

To help address these limitations, we developed chewR, an open‐source R package designed to quantify masticatory performance through automated image analysis of the two‐color gum mixing task. chewR processes scanned gum images by automatically isolating the gum region, extracting color information, and computing masticatory performance metrics without reliance on manual image‐processing steps. The primary aim of this study was to evaluate the convergent validity of chewR by comparing masticatory performance metrics derived from chewR with masticatory performance derived from ViewGum, an established image‐based method for two‐color gum analysis (Halazonetis et al. 2013; Schimmel et al. 2015). Our secondary aim was to examine associations between masticatory performance derived from chewR and other oral processing behaviors including objectively measured eating rate, self‐reported eating rate, and stimulated salivary flow. Specifically, we hypothesized that greater masticatory performance would be associated with higher eating rate and greater stimulated salivary flow.

## 2 Methods

### 2.1 Participant Recruitment and Screening

Prospective participants were recruited from an opt‐in database maintained by the Sensory Evaluation Center at Penn State. This pool consists of individuals who reside in the State College, Pennsylvania area and have previously indicated they are interested in participating in chemosensory and food‐related research. Eligibility was assessed using an online screening questionnaire via Compusense (Compusense Inc. 2024). Inclusion criteria were: between the ages of 22–60, fluent in English, in generally good health, willing to consume raw carrots, willing to chew and spit out gum, willing to provide a saliva sample, willing to avoid alcohol the day before and day of testing, and willing to complete all study procedures.

Individuals were excluded if they wear full or partial dentures, lacked a full set of natural teeth, or reported chewing disabilities, recent oral pain, or dental work within the past 30 days. No clinical oral examination, independent dental assessment, or validated oral health screening tools were used here, meaning that any exclusions based on oral health were based solely on self‐report by participants. Additional exclusion criteria included difficulty swallowing or a history of choking, reported taste or smell issues, and food allergies or intolerances to the test foods. Individuals were also excluded if they were pregnant or breastfeeding, had a diagnosis of depression, an eating disorder, or health conditions affecting appetite, or used medications known to alter appetite, pain perception, taste, or smell within the past 3 months. Individuals who were currently or had recently used nicotine (past 30 days) or were currently taking prescription pain medication were excluded. Last, students, staff, and faculty in Nutritional Sciences, Food Science, or Psychology were excluded to minimize potential bias associated with informed speculation about study aims.

The sample size was determined based on feasibility for this methods validation study; no formal a priori power analysis was conducted. All data were collected between September and October 2025 on the main Penn State campus. Participants completed a single laboratory visit between 10 am and 4 pm. All participants provided informed consent prior to their test session and they received a small cash incentive for their time. All study procedures were approved by The Pennsylvania State University Institutional Review Board (STUDY00027802).

### 2.2 Study Overview

Upon arrival at the laboratory, participants completed a masticatory performance task using a two‐color gum test. After this task, participants consumed 100 mL of water and waited 3 min. Participants then completed the carrot test to quantify eating rate with a real food. Following the carrot test, participants took a 5 min break, during which they rinsed their mouth with water to help remove any residual food prior to salivary collection. Last, participants completed a stimulated salivary flow task and the test session ended with a brief set of questionnaires. This study was not meant to be a clinical trial, nor test a clinical sample, so it was not preregistered. Still, a completed STROBE checklist is provided in the Supporting Information for interested readers.

### 2.3 Measures

#### 2.3.1 Masticatory Performance

The specific terminology used to describe chewing function varies across the dental, aging, nutrition, eating behavior, and oral processing literatures and includes terms such as masticatory performance, chewing performance, masticatory efficiency, and chewing efficiency. Here, we operationally define masticatory performance as the degree of color mixing that is achieved after a fixed number of chewing cycles, acknowledging that other phrasing or methods are common. Specifically, we measured masticatory performance using a two‐color gum mixing task with commercially available Hubba Bubba Watermelon Strawberry gum that consists of red and green color layers. Each piece of gum was standardized to a mass of 4.1 g±0.2 and cut at the midpoint to an approximate length of 11.0 mm. All gum pieces were prepared the afternoon prior to the test day and were stored at room temperature in sealed containers until use to prevent them from drying out.

Participants completed a series of masticatory performance tasks consisting of four trials of 5, 10, 15, or 20 chews. The order of the number of chews was counterbalanced using a Williams design, where participants completed the 5‐, 10‐, 15‐, and 20‐chew trials in different sequences rather than all participants completing the trials in the same order. Participants received the following instructions: “Place the entire piece of gum in your mouth on your preferred chewing side and chew for the number of chews displayed on the screen.” After completing the specified number of chews, participants expectorated their gum into a provided container. A one‐minute break was included between chewing tasks.

Following collection of all gum samples, all samples were immediately prepared for imaging while still soft and hydrated. Specifically, the gum was placed in the center of a small plastic bag, positioned on a large metal sheet, and flattened using a rolling pin to a uniform thickness of 1.0 mm using a custom jig. The bag was flipped and flattened again. The resulting flattened gum inside the bag was scanned on a CanoScan LiDE 300 flatbed scanner at 600 dpi resolution with the scanner lid closed. Each sample was scanned once per side to obtain front and back images. Both images were used for subsequent processing and analysis in ChewR and ViewGum. The final masticatory performance variables used in subsequent analyses were ViewGum score, chewR Raw Score, and chewR Masticatory Performance Score (details below). This procedure was adapted from two‐color gum mixing protocols described previously by Schimmel and colleagues (Schimmel et al. 2007, 2015), with the primary differences being the use of commercially available gum and chew counts being displayed on a screen rather than being counted by the researcher.

#### 2.3.2 Eating Rate

Eating rate was measured using the carrot test described previously by Tang and colleagues (Tang et al. 2025). Here, all carrots were standardized to a mass of 15.0 g±1.0 and dimensions of approximately 11.0 cm in length and 1.0 cm in thickness. Carrots were cut and stored in a refrigerator the day prior to testing. Carrots were removed from the refrigerator approximately 15 min before each test session. Prior to beginning this task, participants were given a three‐minute break during which they consumed 100 mL of water. This step was implemented to minimize any carryover effects from the previous masticatory performance task with the gum samples. After this short break, participants were served two carrots with the following instructions: “Please consume both carrots as you normally would. Eat at your natural pace until finished.”

Eating behavior was recorded using a Logitech C920 webcam mounted slightly below eye level to capture facial orientation and swallowing. The recordings were collected via OBS Studio at 1080p and 30 frames per second. Participants could see the camera but could not see themselves during the recording. This is the same approach our group has used previously (Harper et al. 2024, 2025). Eating Rate was calculated by dividing the grams of carrots consumed by the eating duration (g/min). This measurement served as an objective measure of eating rate.

#### 2.3.3 Stimulated Salivary Flow

Before measuring salivary flow, participants were given another 5 min break during which they were instructed to rinse their mouth thoroughly and spit out the water into a provided cup until no carrot residue remained. To assess stimulated salivary flow, participants were asked to chew a piece of Parafilm for 5 min, consistent with prior research (Ma et al. 2025). Exact instructions were: “Chew the Parafilm continuously for five minutes and spit every 30 s into the collection tube.” A timer was displayed on the screen to help guide the participant. The total mass of saliva was recorded by weighing the collection tube before and after measurement. The final salivary‐flow variable was computed as grams of saliva secreted per minute (g/min).

#### 2.3.4 Self‐Reported Eating Rate and Chewing Function

Self‐reported eating rate was measured using a single visual analogue scale (VAS). Participants were asked: “Compared to other people of your age and sex, how would you describe the rate at which you eat a meal?” Responses were recorded on a 100 point VAS anchored at extremely slow and extremely fast. Higher values indicate faster self‐perceived eating rate. Next, participants completed the Chewing Function Questionnaire that is designed to assess self‐reported chewing ability during everyday eating (Peršić et al. 2013).

### 2.4 chewR Pipeline

#### 2.4.1 chewR Overview

**FIGURE 1:**
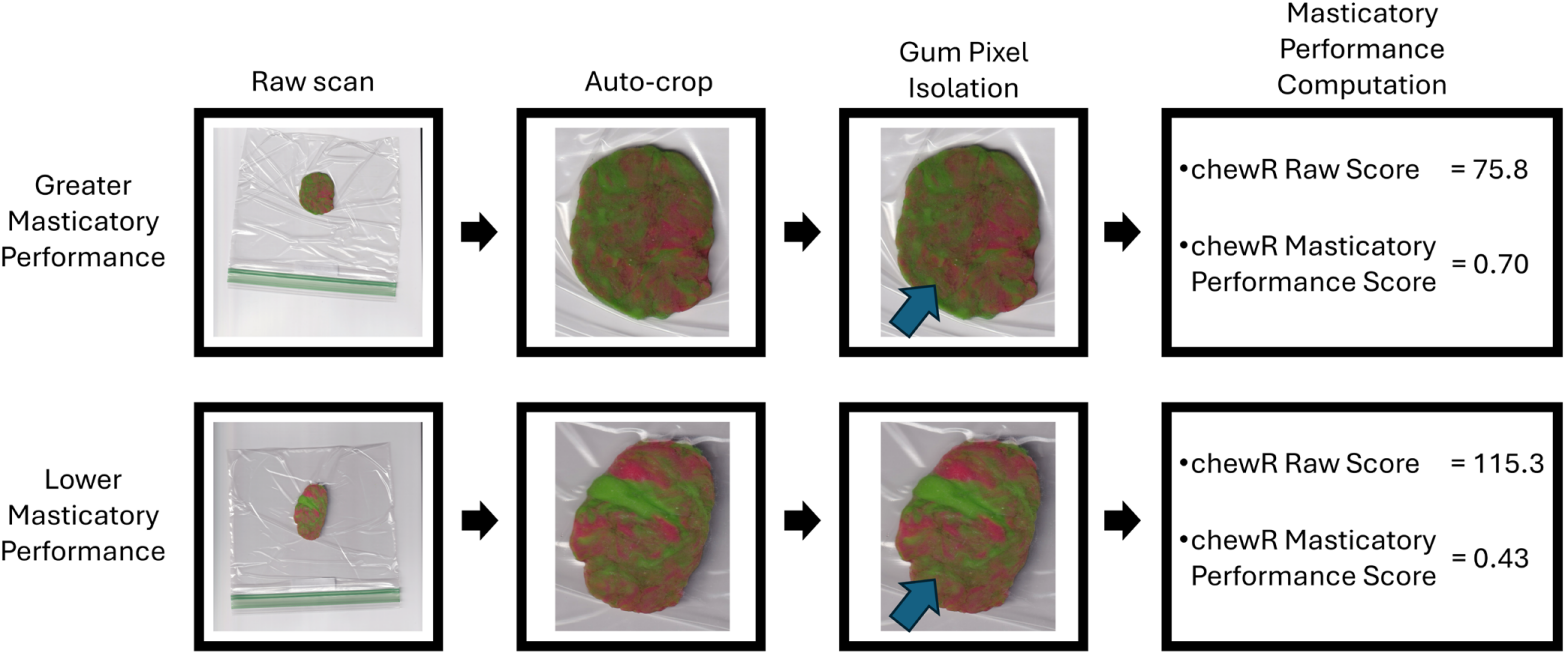
Overview of the chewR processing pipeline. The raw scanned image of a chewed two‐color gum sample contained within a plastic bag is auto cropped and the gum region is isolated for analysis. The masticatory performance variables are computed as raw or transformed into the chewR Masticatory Performance score. The top row illustrates greater masticatory performance and the bottom row illustrates lower masticatory performance. Images are shown for illustrative purposes and were resized for figure presentation.

Masticatory Performance was computed using *chewR*, a novel open‐source R package specifically developed for computing masticatory performance through automated analysis of two‐color gum images. The chewR pipeline processes scanned gum images in batches by auto‐cropping images to isolate the gum region, isolates the gum pixels, extracts the hue, and computes several quantitative masticatory performance variables (Figure 1). The subsequent sections outline these processes in more specific detail.

#### 2.4.2 Filename Metadata Extraction

Image filenames are based on the participant identifier, number of chews, and scan side (front/back). These aspects were parsed programmatically based on the file name to ensure each image was assigned to the correct participant and chew condition prior to analysis: as a hypothetical example, 13_20chews_side1 would be participant ID 13, chewed 20 times, and is the front side of the gum while 13_20chews_side2 would be the back side of the same gum sample.

#### 2.4.3 Autocropping and Gum Region Isolation

To ensure analysis was restricted to the gum sample rather than the entire image, each image is automatically cropped to isolate the gum region. This autocropping step identifies the gum as the primary color‐containing region within the scan and removes excess background and unused scan space. This is particularly relevant as zip top bags may contain color that interferes with image analysis. If the user does not want to use the autocropping function, the user can manually crop images to isolate the gum sample prior to analysis.

#### 2.4.4 Background Removal and Pixel Extraction

After autocropping, non‐gum background pixels were removed using a color‐thresholding filter and only pixels corresponding to the gum were retained for analysis. This step excluded the white scanner background and other non‐gum regions, ensuring that the subsequent color analysis reflected gum pixels only. For replication in future studies, scans should be collected against a uniform white background (e.g., white paper on the scanner bed) to facilitate reliable background exclusion.

#### 2.4.5 HSV Conversion and Hue Extraction

Pixels from the resulting extraction were converted from RGB to HSV color space and the hue values were extracted for each gum pixel. Hue values were used because they reflect color identity independent of brightness, making them suitable for quantifying the degree of color mixing of the gum sample.

#### 2.4.6 Computation and Scaling of Masticatory Performance Variables

Masticatory performance was quantified as the dispersion of the hue values across the gum pixels. This raw variable is operationalized as the standard deviation of hue (H_SD). Lower H_SD values indicate greater gum color homogeneity (greater mixing) and therefore greater masticatory performance, whereas higher H_SD values indicate greater gum heterogeneity (poorer mixing) and therefore poorer masticatory performance. The chewR package computes H_SD separately for each scan side (front and back) and also computes a combined H_SD value by pooling hue values across both sides. This combined value serves as the primary raw measure of masticatory performance and is named *chewR Raw Score* for convenience.

For enhanced interpretability, an additional chewing‐efficiency score was then derived by applying a linear transformation to H_SD: [2*(295‐H_SD)/(295)‐0.79]. This transformation preserves relative differences between samples while producing a score where higher values correspond to greater masticatory performance, yielding values bounded between 0 and 1. This variable is called *chewR Masticatory Performance Score* for convenience. Here, constants of 295 and 0.79 were chosen to yield values in the 0–1 range for the H_SD distribution observed from the 20‐chew condition in the present dataset. In adults with intact dentition, this transformation yielded a minimum value of 0.42 and a maximum value of 0.85, leaving room for values that may be observed in less efficient chewers or other populations without intact dentition. We anticipate values may be more extreme in other populations. Alternative scaling constants or calibration‐based normalization may be required for different gum colors, scanners, or imaging conditions.

#### 2.4.7 Replication and Comparison Across Studies

To facilitate replication and comparison across studies, researchers should aim to use the same gum sample and scanner model (e.g., 600 dpi with white paper on the scanner bed). Under these conditions, both the chewR Raw Scores and the chewR Masticatory Performance Score variables are comparable across studies. However, when different gum colors or other imaging setups are used, both masticatory performance variables should be interpreted within‐study and not across studies. Future work will focus on expanding reference datasets and developing standardized calibration approaches to facilitate broader comparability across studies, including clinical populations lacking intact dentition.

#### 2.4.8 chewR Pipeline Processing Code in R Tutorial

**FIGURE 2:**
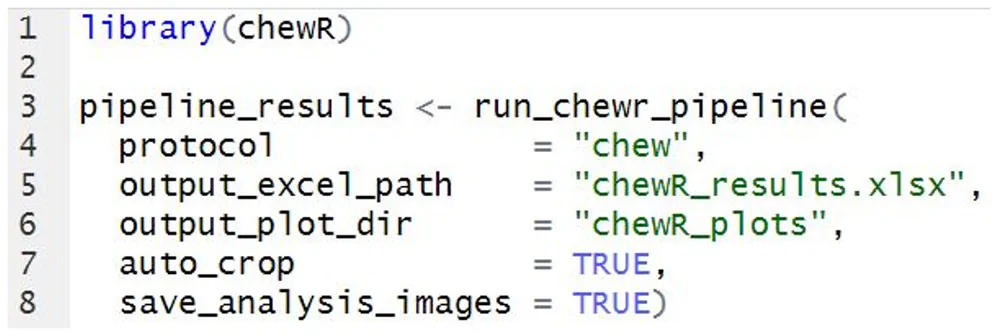
Example of running the chewR pipeline. The user specifies a directory (folder) of scanned two‐color gum images. The pipeline automatically processes all images and returns the chewing efficiency variables.

An example implementation of the chewR pipeline is shown in Figure 2. In brief, the user specifies a directory (folder) containing scanned gum images and runs a single pipeline function that automatically processes all images in a batch. This function parses filename metadata, optionally auto‐crops images to isolate the gum region, removes background pixels, extracts hue values, and computes the masticatory performance variables for each image.

#### 2.4.9 chewR Availability and Licensing

The chewR package is open‐source and released under the MIT License. It is freely available for use without restriction and does not require additional permission. The package, along with documentation and a step‐by‐step tutorial, is available at: https://github.com/JohnLongPhD/chewR.

### 2.5 Validation of chewR

To evaluate the convergent validity of chewR, masticatory performance scores derived from chewR were correlated with masticatory performance scores derived from ViewGum, an established image‐based method for assessing two‐color gum mixing. ViewGum analyses were performed independently by two operators on the same scanned images used for chewR to ensure that all comparisons reflected differences in the analytical approach rather than differences in samples or imaging conditions. Briefly, ViewGum transforms the gum image into hue saturation and intensity color space. The user then isolates the gum region in software, and hue values are calculated from the pixels within the isolated region. For each gum sample, ViewGum produces a ViewGum Score reflecting the degree of color mixing across chewed gum, where lower scores equate to greater masticatory performance. chewR Raw Score and chewR Masticatory Performance Score were computed as described above.

### 2.6 Data Analysis

Although 65 participants completed the study, duplicate observations were excluded because they violate the independence assumption of Pearson's correlation. Participant trials with documented errors in carrot eating measurement were also excluded. In cases where a participant repeated the carrot test following an invalid trial, the valid replacement observation was retained. This resulted in a final dataset of 47 participants.

The demographic characteristics of the sample were assessed and summarized via means, standard deviations, frequencies, and percentages. A linear mixed‐effects model was used to determine the dose–response relationship between chewR Masticatory Performance Score and chews. The outcome was chewR Masticatory Performance Score, with chews and participant sex as fixed effects and participant ID as a random intercept. All subsequent masticatory performance analyses were conducted using data from the 20‐chew condition only, which is consistent with prior literature that has reported this chew count as optimal for assessing masticatory performance (Buser et al. 2018; Gonçalves et al. 2021).

An intraclass correlation coefficient (ICC) and its 95% confidence interval were calculated to assess the inter‐rater reliability of the ViewGum Score using a two‐way random‐effects, absolute‐agreement, single‐measurement model (ICC[2,1]). For subsequent convergent validity analyses, the mean ViewGum Score across the two raters was used.

Convergent validity of chewR was assessed using Pearson's correlations between ViewGum Score and both the chewR Raw Score and chewR Masticatory Performance Score. Pearson's correlations were also conducted to examine associations between masticatory performance and other oral processing behaviors, including correlations between chewR Masticatory Performance Score and eating rate, self‐reported eating rate, and stimulated salivary flow. Additional Pearson's correlations were calculated to examine relationships between stimulated salivary flow and eating rate, as well as stimulated salivary flow and self‐reported eating rate (Buser et al. 2018; Gonçalves et al. 2021). All statistical analyses were conducted using R. The data and code used to analyze and visualize the data are available via Open Science Framework at https://osf.io/2pjnu.

## 3 Results

### 3.1 Participant Characteristics

The final sample included 47 participants. The demographic characteristics of the sample are reported in Table 1.

**TABLE 1:** Participant characteristics (*n* = 47).

	Total (*n*)	Percentage (%)	
*Sex*			
Male	19	40	
Female	28	60	
*Race/Ethnicity*			
White	39	83	
All other races	8	17	
*Education*			
No degree	7	15	
Degree	40	85	
*Income*			
< $100 k USD	25	53	
≥ $100 k USD	21	45	
Prefer not to answer	1	2	

	Mean	SD	Range
Age (year)	39.0	12.4	20–60
Height (m)	1.69	0.09	1.55–1.96
Weight (kg)	77.0	15.8	110–285
Body Mass Index (kg/m^2^)	26.8	5.4	18.6–43.3

*Note:* All data presented above 1 is self‐reported.

### 3.2 Dose–Response Relationship Between Number of Chews and chewR Masticatory Performance Score

**FIGURE 3:**
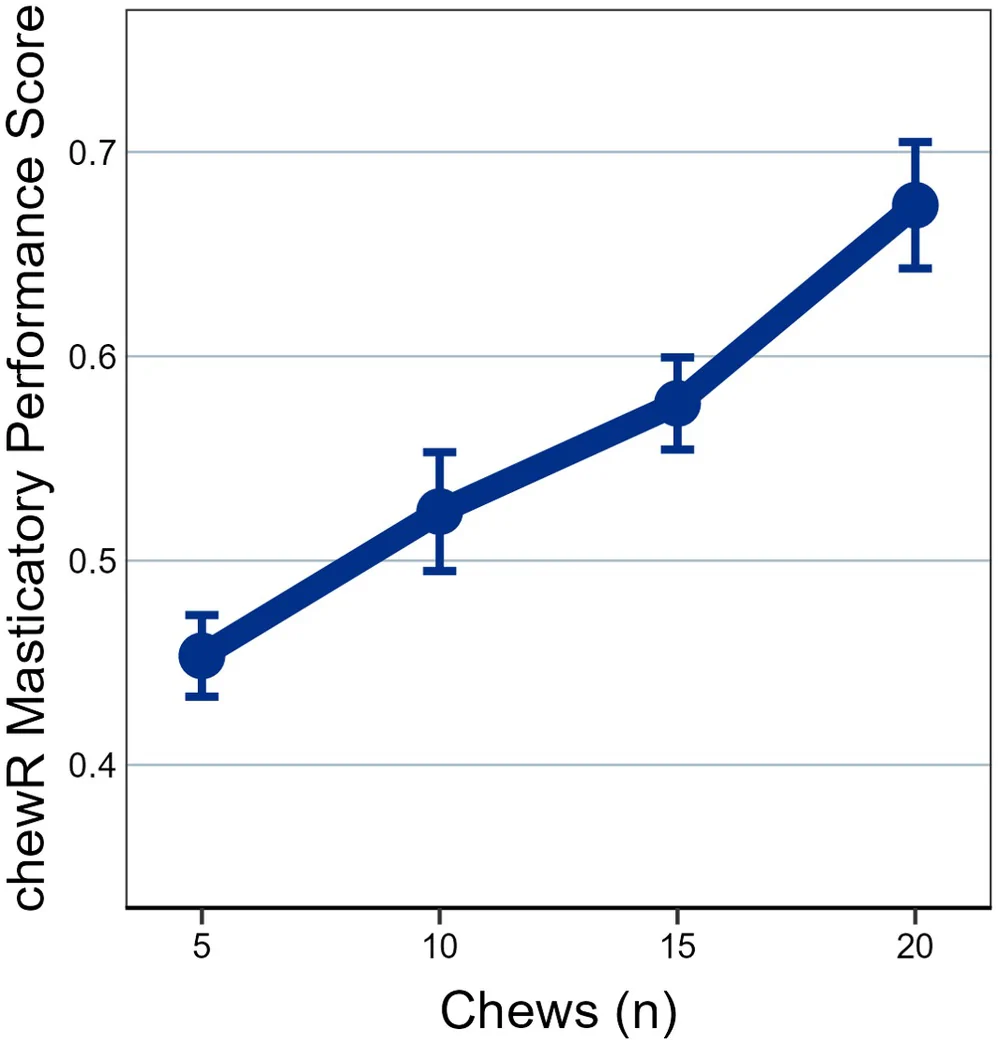
Dose response relationship between chews and chewR Masticatory Performance Score. chewR Masticatory Performance Score increased across the chew conditions. Points represent the mean chewR Masticatory Performance Score at each chew condition. The vertical error bars represent the 95% confidence intervals. The connecting line depicts an increase in masticatory performance across the chew conditions.

As expected, chewR Masticatory Performance Score increased in a dose dependent manner: each additional chew was associated with a 0.014‐unit increase (SE = 0.001, *p* < 0.0001) in chewR Masticatory Performance Score. Mean ± SD scores increased from 0.45 ± 0.07 at 5 chews to 0.52 ± 0.10 at 10 chews, 0.58 ± 0.08 at 15 chews, and 0.67 ± 0.11 at 20 chews. These data are visualized in Figure 3.

### 3.3 Inter‐Rater Reliability of ViewGum

Inter‐rater reliability of ViewGum Score was 0.999 (95% CI: 0.998–1.000, *p* < 0.0001), indicating excellent (near‐perfect) agreement between two independent operators of the software.

### 3.4 Preliminary Validation of chewR

**FIGURE 4:**
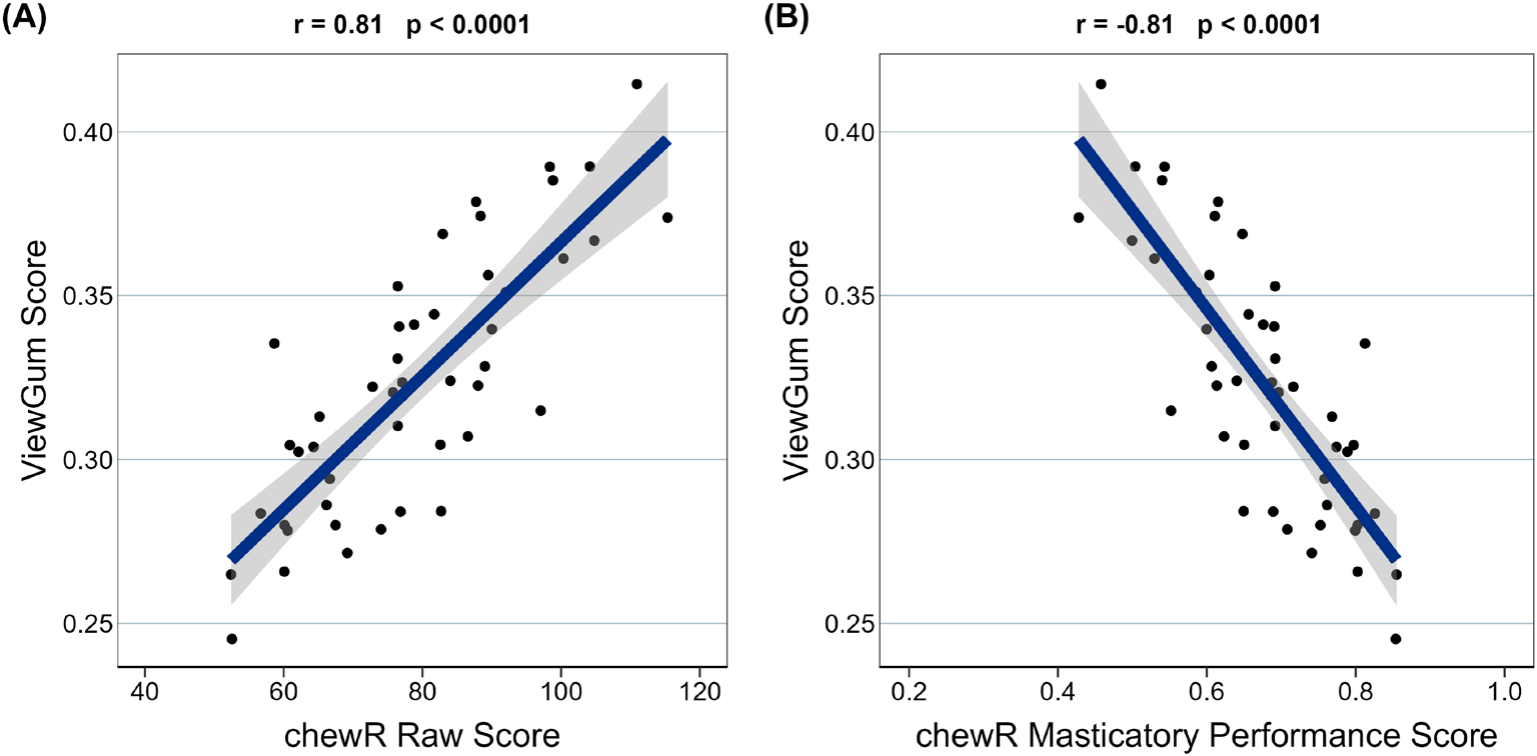
Convergent validity of chewR masticatory performance variables with ViewGum. Masticatory performance computed using the chewR packages strongly correlates with masticatory performance computed using ViewGum. (A) Association between the chewR Raw Score and ViewGum Score. (B) Association between the chewR Masticatory Performance Score and ViewGum Score. Lower chewR Raw Scores and lower ViewGum Scores reflect greater masticatory performance, whereas higher chewR Masticatory Performance Scores indicate greater masticatory performance. The correlations are visualized with individual data points, accompanied by a linear trend line and their 95% confidence interval.

Masticatory performance computed using the chewR package demonstrated strong convergent validity with the proprietary ViewGum software. Specifically, the chewR raw score was strongly correlated with ViewGum Score (*r* = 0.81, *p* < 0.00001) and the chewR Masticatory Performance Score was similarly strongly correlated with the ViewGum Score in the expected negative direction (*r* = −0.81, *p* < 0.0001). These data are visualized in Figure 4.

### 3.5 Exploratory Analyses: Associations Between Masticatory Performance, Eating Rate, and Salivary Flow

**FIGURE 5:**
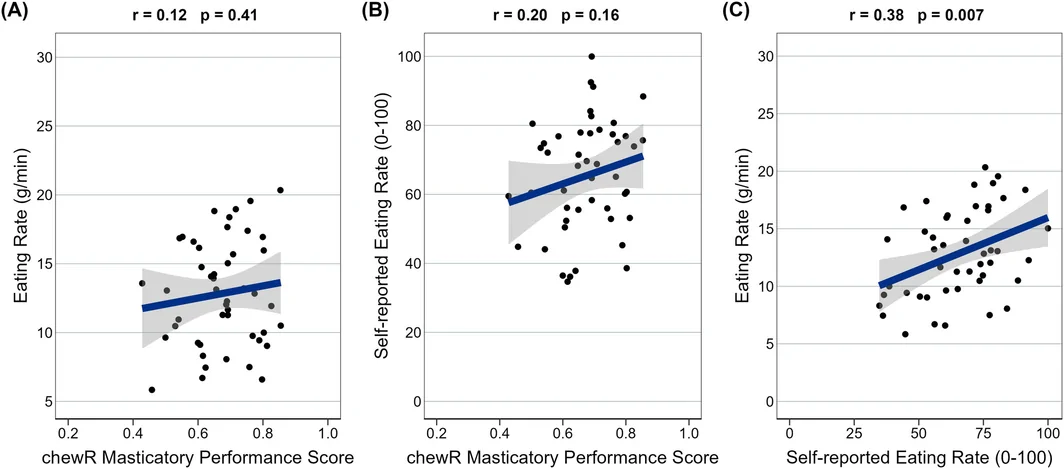
Associations between masticatory performance, eating rate, and self‐reported eating rate. (A) Association between the chewR Masticatory Performance Score and objectively measured eating rate from the carrot test. (B) Association between the chewR Masticatory Performance Score and self‐reported eating rate. (C) Association between objectively measured eating rate and self‐reported eating rate. The correlations are visualized with individual data points, accompanied by a linear trend line and their 95% confidence interval.

The chewR Masticatory Performance Score was not meaningfully associated with objectively measured eating rate derived from the carrot test (*r* = 0.12, *p* = 0.41), or with self‐reported eating rate (*r* = 0.20, *p* = 0.16). As expected, objectively measured eating rate (i.e., carrot test) was moderately correlated with self‐reported eating rate (*r* = 0.38, *p* = 0.007). These data are visualized in Figure 5.

**FIGURE 6:**
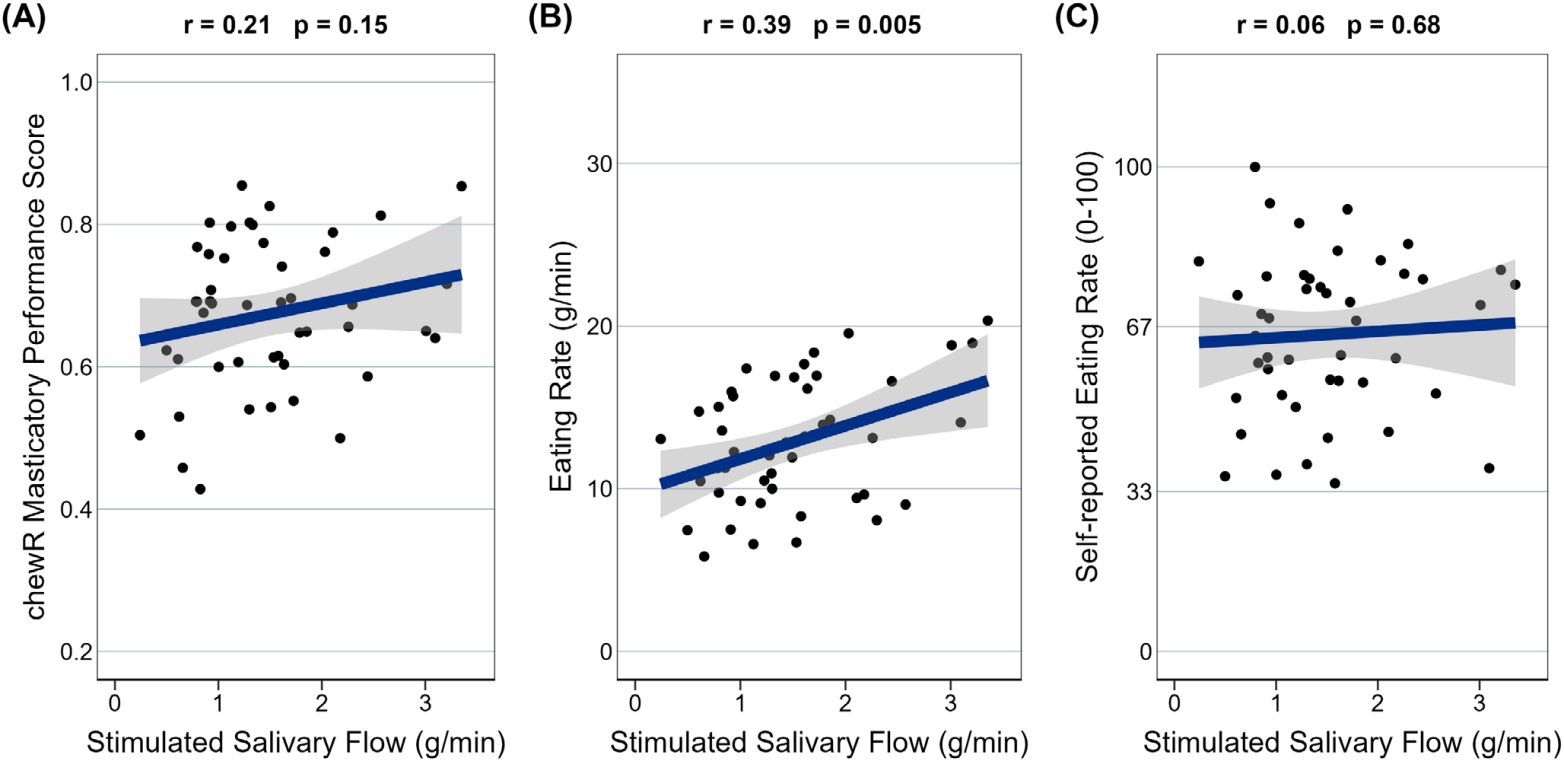
Associations between stimulated salivary flow, masticatory performance, and eating rate. (A) Association between stimulated salivary flow and the chewR Masticatory Performance Score. (B) Association between stimulated salivary flow and objectively measured eating rate derived from the carrot test. (C) Association between stimulated salivary flow and self‐reported eating rate. The correlations are visualized with individual data points, accompanied by a linear trend line and their 95% confidence interval.

**FIGURE 7:**
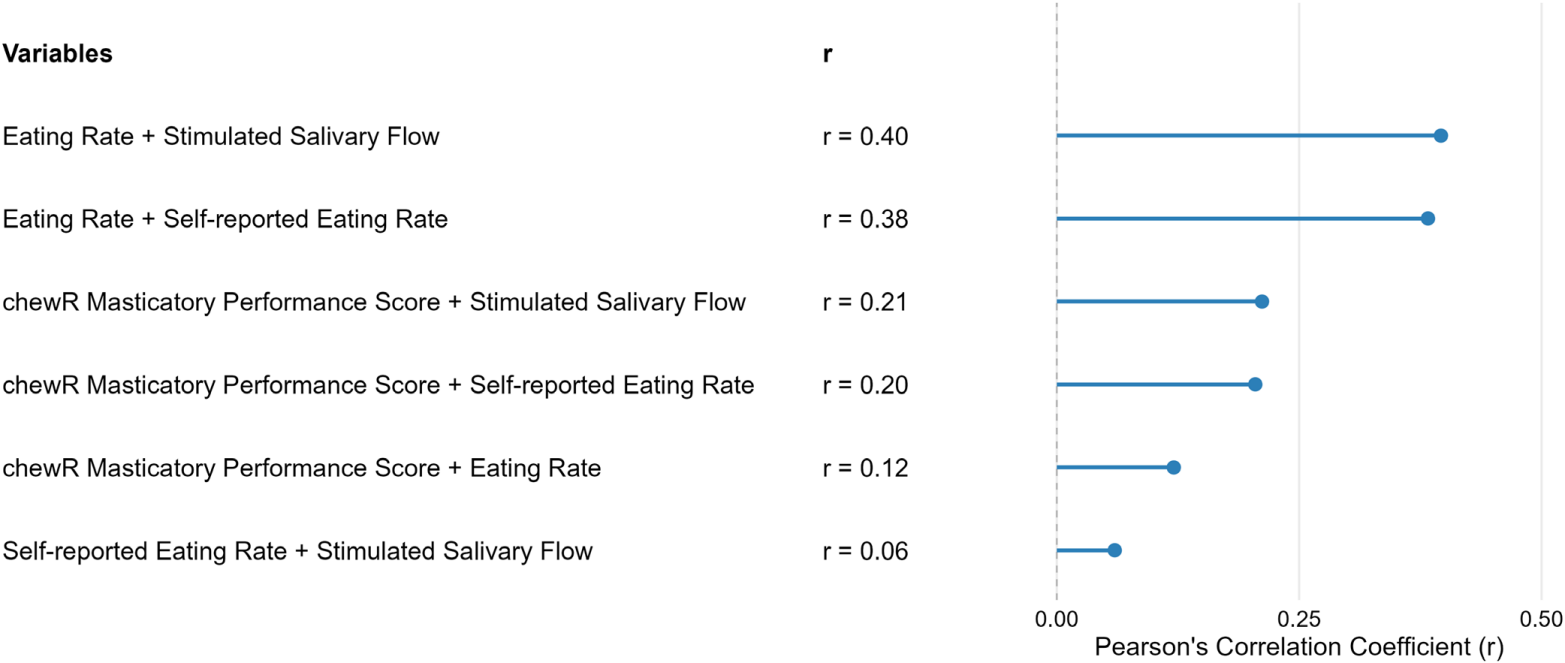
Pairwise correlations among oral processing variables. Pearson's correlation coefficients between eating rate, self‐reported eating rate, stimulated salivary flow, and chewR Masticatory Performance Score. Each point represents a correlation coefficient with horizontal lines indicating the magnitude of the relationship.

Stimulated salivary flow was not meaningfully associated with the chewR Masticatory Performance Score (*r* = 0.21, *p* = 0.15), nor with self‐reported eating rate (*r* = 0.06, *p* = 0.68). In contrast, stimulated salivary flow showed a modest correlation with objectively measured eating rate derived from the carrot test (*r* = 0.39, *p* = 0.005). These relationships are visualized in Figure 6. Pairwise associations between eating rate, self‐reported eating rate, salivary flow, and chewR Masticatory Performance Score are visualized in Figure 7. All analyses reported in this section were exploratory outcomes, so lack of evidence should be interpreted cautiously as the study was not powered for these secondary outcomes.

## 4 Discussion

Here we describe chewR, an open‐source R package for quantifying masticatory performance using automated image analysis of the two‐color gum mixing task. Masticatory performance metrics derived from chewR showed strong convergent validity with values obtained from ViewGum, supporting chewR as an open‐source tool for computing masticatory performance from two‐color gum images. Although ViewGum and chewR both quantify masticatory performance from color mixing in images of chewed gum, the two methods are not identical image processing pipelines. Hue‐based metrics can be sensitive to preprocessing decisions such as isolating the gum region, background removal, pixel filtering, and scanner artifacts. Therefore, the remaining unexplained variation likely reflects differences in image (pre‐)processing or operator choices rather than evidence that the methods measure different underlying chewing constructs. These findings support chewR as a reproducible and accessible tool for quantifying masticatory performance and provide a foundation for examining its role in oral processing and eating behavior.

In addition to examining the convergent validity of chewR, this study examined how masticatory performance relates to other oral processing behaviors, including eating rate and salivary flow as secondary outcomes. Here, masticatory performance measured using chewR was weakly associated with objectively measured eating rate derived from the carrot test, as well as self‐reported eating rate. These findings suggest masticatory performance and eating rate may capture distinct aspects of oral processing, which aligns with other recent research that found no significant association between masticatory performance and eating rate (Zannidi et al. 2025). While eating rate is a temporal measurement of food intake, masticatory performance reflects the effectiveness of food breakdown for a given number of chews. This distinction suggests that eating rate and chewing efficiency are distinct oral processing behaviors that may uniquely contribute to eating behavior.

Salivary flow represents another core aspect of oral processing that may affect chewing and swallowing. In the present data, stimulated salivary flow was moderately correlated with objectively measured eating rate but was not correlated with masticatory performance. These results suggest that salivary flow may influence eating behavior through mechanisms distinct from masticatory performance (e.g., sample wetting or bolus formation). Consistent with this interpretation, other recent research finds no significant association between stimulated salivary flow and masticatory performance (Zannidi et al. 2025). However, that study demonstrated that stimulated salivary flow was associated with bolus salivary uptake, which in turn was related to masticatory performance, suggesting that saliva may influence oral processing by facilitating bolus formation rather than by enhancing mechanical mastication. Taken together, these findings suggest that salivary flow may influence oral processing through pathways related to bolus formation and lubrication, rather than through direct effects on masticatory performance, with the additional caveat that such effects may also vary across food type (i.e., soft versus hard, wet versus dry).

The chewR package computes several masticatory performance variables, including a raw score and a transformed score. The chewR Raw Score is based on the dispersion of hue values within the chewed gum sample and is operationalized as the standard deviation of hue. This traditional operationalization is mathematically sound where lower values reflect greater color homogeneity and therefore greater masticatory performance. While this is consistent with other image‐based approaches (Halazonetis et al. 2013), it can be counterintuitive when interpreting results. For example, a positive correlation between the chewR Raw Score and energy intake would indicate that participants with lower masticatory performance consumed more energy, whereas a significant positive correlation between the chewR Masticatory Performance Score and energy intake would indicate that participants with greater masticatory performance consumed more energy. Thus, the transformed masticatory performance variable provided in the current version of the chewR package preserves the relative difference between samples while improving interpretability and facilitating clearer communication of results. Accordingly, we recommend using the chewR Masticatory Performance Score for analyses and reporting of results. However, the raw metric is retained within the package to support flexibility and reproducibility across studies and populations.

Future directions include extending validation of chewR across a broader range of gum types, including variations in color combinations, to broaden the generalizability of chewR. Another direction involves evaluating chewR using alternative imaging methods, such as using a smartphone, to increase ease of measurement and accessibility outside controlled laboratory settings; still, we note that the scanner used here is widely available and relatively low cost. Other groups have demonstrated the utility of using high‐resolution smartphone cameras for measuring masticatory performance (Buser et al. 2018; Fankhauser et al. 2020). This may facilitate measuring populations outside a university setting by reducing reliance on laboratory‐based equipment. Future work should evaluate chewR in broader communities and clinical populations, such as prosthodontic and geriatric populations. This would improve generalizability, as the present sample was recruited from a university community that was predominantly white and educated. A related future direction is measuring chewR in populations with poor oral health or compromised dentition, which should offer additional insight into how masticatory performance interacts with other oral processing behaviors across more diverse populations. Such studies should also include dental status variables that are determinants of masticatory performance, such as number of remaining teeth, number of occlusal contacts, and Angle classification. Finally, applying chewR in other oral processing and eating behavior paradigms may help clarify how masticatory performance is related to distinct aspects of oral processing and eating behavior.

Several limitations should be considered when interpreting these findings. The analysis of convergent validity described here compares chewR with ViewGum using the same scanned gum images. Therefore, the strong association should be interpreted as evidence of convergence with an established image‐based method rather than independent validation against another, more mechanistic, gold standard method. Future studies should compare chewR scores against other methods (e.g., particle size distribution of sieved silicone cubes after chewing). We should also note that the chewR package relies on hue‐based dispersion to quantify color mixing, which assumes sufficient color contrast and stable hue separation between the two colors in the gum. Different color combinations, such as blue‐pink or red‐green, may produce different ranges and distributions of hue values, potentially influencing the magnitude of the raw masticatory performance metric. Further, the transformed chewR Masticatory Performance Score applies to a linear scaling constant derived from raw values found in the present dataset. This transformation improves interpretability but is not universally calibrated and may require adjustment if different gum colors, scanners, or imaging conditions are used. Under such conditions, both raw and transformed chewR metrics should be interpreted relatively within a study rather than compared directly across studies. Thus, the transformed score is intended to improve interpretability within the current scaling parameters, while the raw score preserves the underlying construct for any potential future calibration efforts as more diverse populations are tested. Also, because chewR is an automated pipeline, repeated processing of the same images in the present dataset produced identical output. Accordingly, we did not assess reliability across repeated sample preparation or scanning. For example, we did not test the consistency of chewR scores when the same gum samples were repositioned and rescanned; this should be revisited in future work. Still, inter‐rater reliability of ViewGum Score between two different operators was excellent, which supports the reliability of ViewGum as a comparator for establishing convergent validity of chewR in the present study. Future work should explicitly test chewR scores and ViewGum scores across repeated measurements, imaging conditions, raters, scanners and cameras, in populations with greater variability in dentition and oral health status, as well as cross validation with other approaches like sieving of Optosil cubes. Here, our measure of eating rate was based on a single standardized test food under controlled laboratory conditions (e.g., Tang's carrot test). Although this approach is based on a validated protocol, future work incorporating a broader range of foods and meals may be needed to draw stronger conclusions regarding potential relationship between masticatory performance and eating rate in more ecologically valid contexts. Last, because the study was not powered to detect small associations among secondary outcomes related to food oral processing, weak correlations between masticatory performance, eating rate, self‐reported eating rate, and salivary flow should be interpreted cautiously and considered primarily as approximate effect‐size estimates for future work.

## 5 Conclusion

In sum, the chewR R package is a user‐friendly open‐source tool for quantifying masticatory performance using automated image analysis of the two‐color gum mixing task. chewR and detailed documentation and step‐by‐step tutorials are freely available on GitHub at https://github.com/JohnLongPhD/chewR. This package facilitates standardized masticatory performance assessment across studies and research settings by eliminating reliance on proprietary software while also eliminating manual image‐processing steps. chewR supports both raw and transformed masticatory performance metrics that aid in the interpretation of the masticatory performance construct.

## Author Contributions

**John W. Long:** project administration, investigation, data curation, formal analysis, visualization, software, writing – original draft, writing – review and editing, methodology. **John E. Hayes:** conceptualization, funding acquisition, writing – review and editing, supervision, resources, methodology. **Elsbeth Akanzinge:** investigation, writing – review and editing.

## Funding

This work in the Sensory Evaluation Center at The Pennsylvania State University was supported by faculty‐controlled discretionary funds, and federal appropriations under the Hatch Act from the USDA National Institute of Food and Agriculture under Project PEN04980 (accession number 7006751) to JEH. The funding agencies did not have a role in any of the following: study design; collection, analysis, and interpretation of data; writing of the report; or decision to submit the article for publication, and the views expressed here belong solely to the authors and do not represent any official U.S. government policy or position.

## Ethics Statement

All participants provided informed consent and were financially compensated for their time. All study procedures were approved by The Pennsylvania State University Institutional Review Board (STUDY00027802).

## Conflicts of Interest

The authors declare no conflicts of interest.

## Supporting information


**Data S1:** STROBE Guidelines.

## Data Availability

The data and code used to analyze and visualize the data are available via Open Science Framework at https://osf.io/2pjnu.
