# Supplementary material for: chewR: An Open‐Source R Package for Quantifying Masticatory Performance With Applications in Oral Processing Research
Source: J Texture Stud. 2026 Sep 1;57(5):e70112. doi: 10.1111/jtxs.70112 (PMC13533583; doi:10.1111/jtxs.70112)
Supplement: Supplementary file 1 [file JTXS-57-e70112-s001.docx]

| **STROBE Guidelines** | | | |
| --- | --- | --- | --- |
| **Section/topic** | **Item number** | **Recommendation** | **Location in manuscript** |
| **Title and abstract** | 1 | Indicate the study’s design with a commonly used term in the title or the abstract. Provide in the abstract an informative and balanced summary of what was done and what was found. | See title/abstract |
| **Introduction: Background/rationale** | 2 | Explain the scientific background and rationale for the investigation being reported. | Introduction: paragraphs 1-2 |
| **Introduction: Objectives** | 3 | State specific objectives, including any prespecified hypotheses. | Introduction: paragraph 3 |
| **Methods: Study design** | 4 | Present key elements of study design early in the manuscript. | Methods: 2.1 and 2.2 |
| **Methods: Setting** | 5 | Describe the setting, locations, and relevant dates, including periods of recruitment, exposure, follow-up, and data collection. | Methods: 2.1 |
| **Methods: Participants** | 6 | Cohort study: give eligibility criteria and sources/methods of participant selection; describe follow-up methods. Case-control study: give eligibility criteria, methods of case/control ascertainment, and rationale for case/control selection. Cross-sectional study: give eligibility criteria and sources/methods of participant selection. For matched studies, give matching criteria and numbers. | Methods: 2.1 |
| **Methods: Variables** | 7 | Clearly define all outcomes, exposures, predictors, potential confounders, and effect modifiers; give diagnostic criteria if applicable. | Methods: 2.3-2.6 |
| **Methods: Data sources/measurement** | 8* | For each variable of interest, give sources of data and details of assessment methods; describe comparability of assessment methods if more than one group. | Methods: 2.3 and 2.4 |
| **Methods: Bias** | 9 | Describe any efforts to address potential sources of bias. | Methods: 2.1-2.4 |
| **Methods: Study size** | 10 | Explain how the study size was arrived at. | Methods: 2.1 |
| **Methods: Quantitative variables** | 11 | Explain how quantitative variables were handled in the analyses; if applicable, describe which groupings were chosen and why. | Methods: 2.3, 2.4, 2.6 |
| **Methods: Statistical methods** | 12 | Describe all statistical methods, including methods used to control for confounding. Describe subgroup/interactions analyses, handling of missing data, sampling strategy if applicable, and sensitivity analyses. | Methods: 2.6 |
| **Results: Participants** | 13 | Report numbers of individuals at each stage of the study, including eligible, included, completing follow-up, and analyzed. Give reasons for nonparticipation at each stage. Consider using a flow diagram. | Results: 3.1 |
| **Results: Descriptive data** | 14 | Give participant characteristics and information on exposures and potential confounders. Indicate number of participants with missing data for each variable. For cohort studies, summarize follow-up time. | Results: 3.1 |
| **Results: Outcome data** | 15 | Cohort study: report numbers of outcome events or summary measures over time. Case-control study: report numbers in exposure categories or summary measures. Cross-sectional study: report outcome events or summary measures. | Results: 3.2-3.5 |
| **Results: Main results** | 16 | Give unadjusted estimates and, if applicable, adjusted estimates with precision, such as 95% confidence intervals. Make clear which confounders were adjusted for and why. Report category boundaries when continuous variables were categorized. If relevant, translate relative risk into absolute risk. | Results: 3.2-3.5 |
| **Results: Other analyses** | 17 | Report other analyses done, such as subgroup analyses, interactions, and sensitivity analyses. | NA |
| **Discussion: Key results** | 18 | Summarize key results with reference to study objectives. | Discussion: paragraphs 1-3 |
| **Discussion: Limitations** | 19 | Discuss limitations, including sources of potential bias or imprecision; discuss direction and magnitude of potential bias. | Discussion: paragraph 6 |
| **Discussion: Interpretation** | 20 | Give a cautious overall interpretation considering objectives, limitations, multiplicity of analyses, results from similar studies, and other relevant evidence. | Discussion: paragraphs 1-5 and concluding paragraph |
| **Discussion: Generalizability** | 21 | Discuss the generalizability or external validity of the study results. | Discussion: paragraphs 6-8 |
| **Other information: Funding** | 22 | Give the source of funding and role of funders for the present study and, if applicable, the original study on which the article is based. | See funding section |
